# Targeting Antitumoral Proteins to Breast Cancer by Local Administration of Functional Inclusion Bodies

**DOI:** 10.1002/advs.201900849

**Published:** 2019-07-24

**Authors:** Mireia Pesarrodona, Toni Jauset, Zamira V. Díaz‐Riascos, Alejandro Sánchez‐Chardi, Marie‐Eve Beaulieu, Joaquin Seras‐Franzoso, Laura Sánchez‐García, Ricardo Baltà‐Foix, Sandra Mancilla, Yolanda Fernández, Ursula Rinas, Simó Schwartz, Laura Soucek, Antonio Villaverde, Ibane Abasolo, Esther Vázquez

**Affiliations:** ^1^ Institut de Biotecnologia i de Biomedicina Universitat Autònoma de Barcelona Bellaterra 08193 Barcelona Spain; ^2^ CIBER de Bioingeniería Biomateriales y Nanomedicina (CIBER‐BBN) C/ Monforte de Lemos 3‐5 28029 Madrid Spain; ^3^ Vall d'Hebron Institute of Oncology (VHIO) Edifici Cellex Hospital Vall d'Hebron 08035 Barcelona Spain; ^4^ Peptomyc S.L. Edifici Cellex Hospital Vall d'Hebron 08035 Barcelona Spain; ^5^ Functional Validation & Preclinical Research CIBBIM‐Nanomedicine Vall d'Hebron Institut de Recerca (VHIR) Universitat Autònoma de Barcelona 08035 Barcelona Spain; ^6^ Drug Delivery & Targeting CIBBIM‐Nanomedicine Vall d'Hebron Institut de Recerca (VHIR) Universitat Autònoma de Barcelona 08035 Barcelona Spain; ^7^ Departament de Biologia Evolutiva Ecologia i Ciències Ambientals Facultat de Biologia Universitat de Barcelona Av. Diagonal 643 08028 Barcelona Spain; ^8^ Departament de Genètica i de Microbiologia Universitat Autònoma de Barcelona Bellaterra 08193 Barcelona Spain; ^9^ Leibniz University of Hannover Technical Chemistry and Life Science Callinstr. 5 30167 Hannover Germany; ^10^ Helmholtz Centre for Infection Research Inhoffenstraße 7 38124 Braunschweig Germany; ^11^ Institució Catalana de Recerca i Estudis Avançats (ICREA) 08010 Barcelona Spain; ^12^ Department of Biochemistry and Molecular Biology Universitat Autònoma de Barcelona Bellaterra 08193 Barcelona Spain

**Keywords:** biofabrication, cancer therapy, functional amyloids, inclusion bodies, protein drug release

## Abstract

Two structurally and functionally unrelated proteins, namely Omomyc and p31, are engineered as CD44‐targeted inclusion bodies produced in recombinant bacteria. In this unusual particulate form, both types of protein materials selectively penetrate and kill CD44^+^ tumor cells in culture, and upon local administration, promote destruction of tumoral tissue in orthotropic mouse models of human breast cancer. These findings support the concept of bacterial inclusion bodies as versatile protein materials suitable for application in chronic diseases that, like cancer, can benefit from a local slow release of therapeutic proteins.

## 1 Introduction

Bacterial inclusion bodies (IBs) are insoluble and discrete particles highly enriched by a single protein species, which deposits as interdigitated amyloidal fibers and nonamyloidal protein forms.^1^ They organize into porous fibrilar networks^2^ that confer mechanical stability, in which native or quasinative protein species are embedded.^3^ These protein clusters, ranging between the nano‐ and micro‐scales, are built up in bacterial cells upon expression of a recombinant gene encoding the IB protein. Upon purification from bacteria, they behave as mechanically stable biomaterials,^4^ easy to be handled in a diversity of platforms, presentations and interfaces. In this regard, IBs have been explored in the context of tissue engineering as soft topographies^4^ since they are nontoxic to mammalian cells.^5^

Being partially composed of functional polypeptides,^6^ IB enzymes have found a role in industrial applications as self‐immobilized catalysts.^7^ In addition, IBs tend to attach and penetrate mammalian cells without any deleterious effect,^8^ via macropynocytosis.^9^ Since part of the functional IB protein is released under physiological conditions (namely cell culture media, intracellular environment upon uptake, or in organic tissues),^9^ IBs act as slow release protein platforms, potentially useful in therapeutic approaches (at both cell or organism levels) for protein replacement therapies or protein drug delivery.^8^, ^10^, ^11^ If the IB protein is targeted to a biological marker, for instance, through the incorporation of a tumor‐homing peptide, IBs can remotely deliver IB proteins through the bloodstream to tumoral tissues upon subcutaneous administration.^12^ The bulk IB material, however, is partially stable in the administration site and it remains detectable for weeks long.^13^ Then, once implanted, IBs act as local protein depots, mimicking the natural amyloid repositories of human hormones in the endocrine system,^14^ found them listed among examples of the expanding catalogues of nontoxic functional amyloids.^15^ In this context, extracellular activities of IB proteins, either attached to IBs or upon release, are responsible for downregulation of cell surface receptor expression (by the display of a peptidic ligand of a cytokine receptor^13^) or for enhanced cell growth (when formed by a growth hormone^8^), through appropriate signaling. Whether and how IB proteins released in vivo can penetrate neighboring cells and interact with intracellular circuits for biological manipulation and therapeutic impact remains poorly explored despite its obvious pharmacological interest.

## 2 Results and Discussion

**Figure 1:**
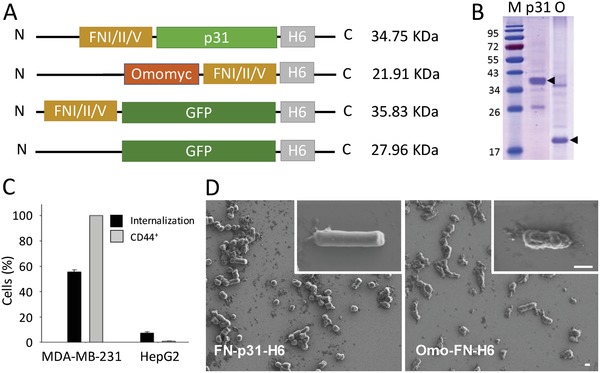
Proteins and protein materials. A) Schematic representation of the fusion proteins FN‐p31‐H6 and Omo‐FN‐H6, indicating the molecular mass of the products. Control proteins used in the study (FN‐GFP‐H6 and GFP‐H6,^26^) are also included. Box sizes are only approximate. B) Coomassie blue staining of a SDS‐PAGE gel loaded with purified proteins. Numbers on the left indicate the molecular masses in kDa of the ladder marker. On the right, arrows indicate the position of the full‐length recombinant proteins. M indicates the molecular marker line, and p31 and O indicate FN‐p31‐H6 and Omo‐FN‐H6 lanes, respectively. C) Internalization of FN‐GFP‐H6 in cultured CD44^+^ MDA‐MB‐231 and CD44^−^ HepG2 cells, measured as the % of green fluorescent cells. Protein was added at 0.5 × 10^−6^ m and exposed to cells for 24 h. The percentage of CD44^+^ cells in each cell line is also indicated as a reference. D) Representative FESEM images of FN‐p31‐H6 and Omo‐FN‐H6 IBs at two different magnifications (zoom in the insets). Magnifications are equivalent in each micrograph pair to allow comparative visualization. Magnification bars represent 500 nm.

To investigate this issue, we have here designed two modular proteins formed by two functional polypeptides (p31 and Omomyc, respectively) involved in cell cycle regulation, with therapeutic potential in antitumoral therapies as tumor‐targeting agents. The p31 protein consists of the C‐terminal fragment of p130cas that has shown the ability to promote apoptosis by disassembling focal adhesion complexes.^16^, ^17^ The Omomyc protein is a Myc dominant negative^18^ extensively validated transgenically and pharmacologically as an antitumoral agent.^19^, ^20^, ^21^ Both proteins were tagged with the tumor‐homing peptide FNI/II/V (FN) which binds CD44, a glycoreceptor that promotes extracellular cell adhesion through hyaluronic acid interaction. CD44 is a well‐recognized tumoral marker, associated to tumor progression and metastasis,^22^, ^23^ and that has been used to identify, along with low expression of CD24, the cancer stem cell population in breast cancer.^24^ Therefore, the use of the tumor‐homing peptide FN seemed appropriate to test targeted nanodepots in a tumor type, the triple negative breast cancer, that currently lacks targeted therapies.^25^ The modular proteins FN‐p31‐H6 and Omo‐FN‐H6 (**Figure** **1**A) were produced in *Escherichia coli* as proteolytically stable full‐length polypeptides (Figure 1B). FN‐GFP‐H6^26^ was also produced as a fluorescent reporter, and this protein was found able to penetrate into 55.6% of CD44^+^ MDA‐MB‐231 cells after 24 h of exposure, but not into CD44^−^ HepG2 cells (7.4%, Figure 1C). This data fully supported the CD44 targeting of the FN segment and the further exploration of FN‐empowered IBs as CD44‐targeted agents. Upon production, an important population of FN‐p31‐H6 and Omo‐FN‐H6 was found in the insoluble cell fraction, aggregated in the form of IBs (Figure 1D). After purification from bacterial cell extracts, p31 and Omomyc IBs were clearly distinguishable in shape and morphology. While the first ones organized in a rod‐shape architecture (≈200 × 1000 nm) with a smooth surface, as previously described for some nonconventional IBs,^27^ Omomyc IBs were smaller (≈200 × 500 nm), exhibiting a rough surface and an ellipsoid geometry that is much more common among bacterial IBs.^28^

**Figure 2:**
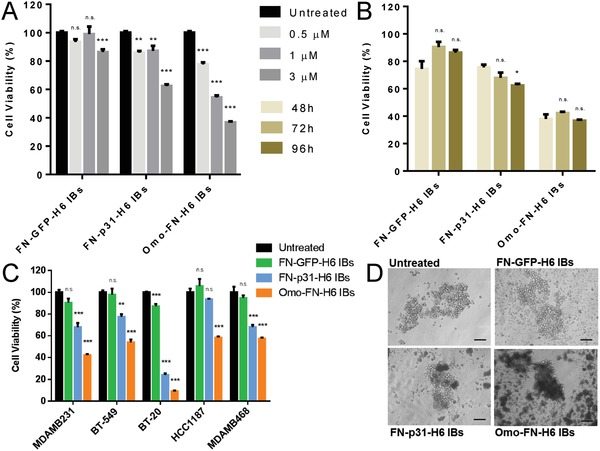
Biological impact of IBs on cultured cells. A) Dose‐dependent loss of MDA‐MB‐231 cell viability upon exposure to IBs for 96 h. At 9 × 10^−6^ m, background reduction of cell viability was observed in cells exposed to GFP‐based IBs (not shown). B) Time‐dependent loss of MDA‐MB‐231 cell viability upon exposure to IBs (3 × 10^−6^ m). C) Killing of different CD44^+^ cell lines by cytotoxic IB (3 × 10^−6^ m), upon exposure for 96 h. D) Representative bright field images of MDA‐MB‐231 tumor‐spheres upon addition of 3 × 10^−6^ m protein and further incubation for 72 h. Qualitative assessment of sphere morphology and integrity when challenged with p31 and Omomyc IBs, compared to an equivalent GFP construct. Magnification bars represent 100 µm. One‐way ANOVA and a post‐hoc Dunnett test was performed comparing all groups to PBS‐treated control cells. *p* > 0.05 (n.s.); *p* < 0.05 (*); *p* < 0.01 (**); *p* < 0.001 (***).

To first explore the potential of these IBs to impact tumor cell biology, cultured breast cancer human MDA‐MB‐231 cells overexpressing CD44^24^, ^29^ were exposed to either p31 or Omomyc IBs and appropriate GFP controls. Both p31 and Omomyc IBs showed a dose‐dependent cytotoxic effect on cell viability, not observed when exposing cells to GFP‐based IBs (**Figure** **2**A). The lack of toxicity associated to exposure to GFP IBs fully confirmed the protein‐associated toxicity of IBs, as the IB material, per se, is not intrinsically cytotoxic.^1^ Moreover, while p31 IBs showed an increasing cell killing potential with longer exposure time, Omomyc IBs showed the highest efficacy already at 48 h post exposure, indicating a faster mechanism of action (Figure 2B). Similar cytotoxic effects of p31 and Omomyc IBs on cell viability were observed in multiple CD44^+^ cell lines,^23^ reaching slightly different extents probably due to intrinsic differences in cell survival mechanisms, while GFP‐based IBs remained nontoxic (Figure 2C). Microscopy images of MDA‐MB‐231 cultured cells exposed to active and nonactive materials, fully confirmed the cytotoxicity promoted by functionalized IBs (Figure 2D). Indeed, functional IBs caused a clear reduction in tumor sphere size when compared to the GFP ones.

**Figure 3:**
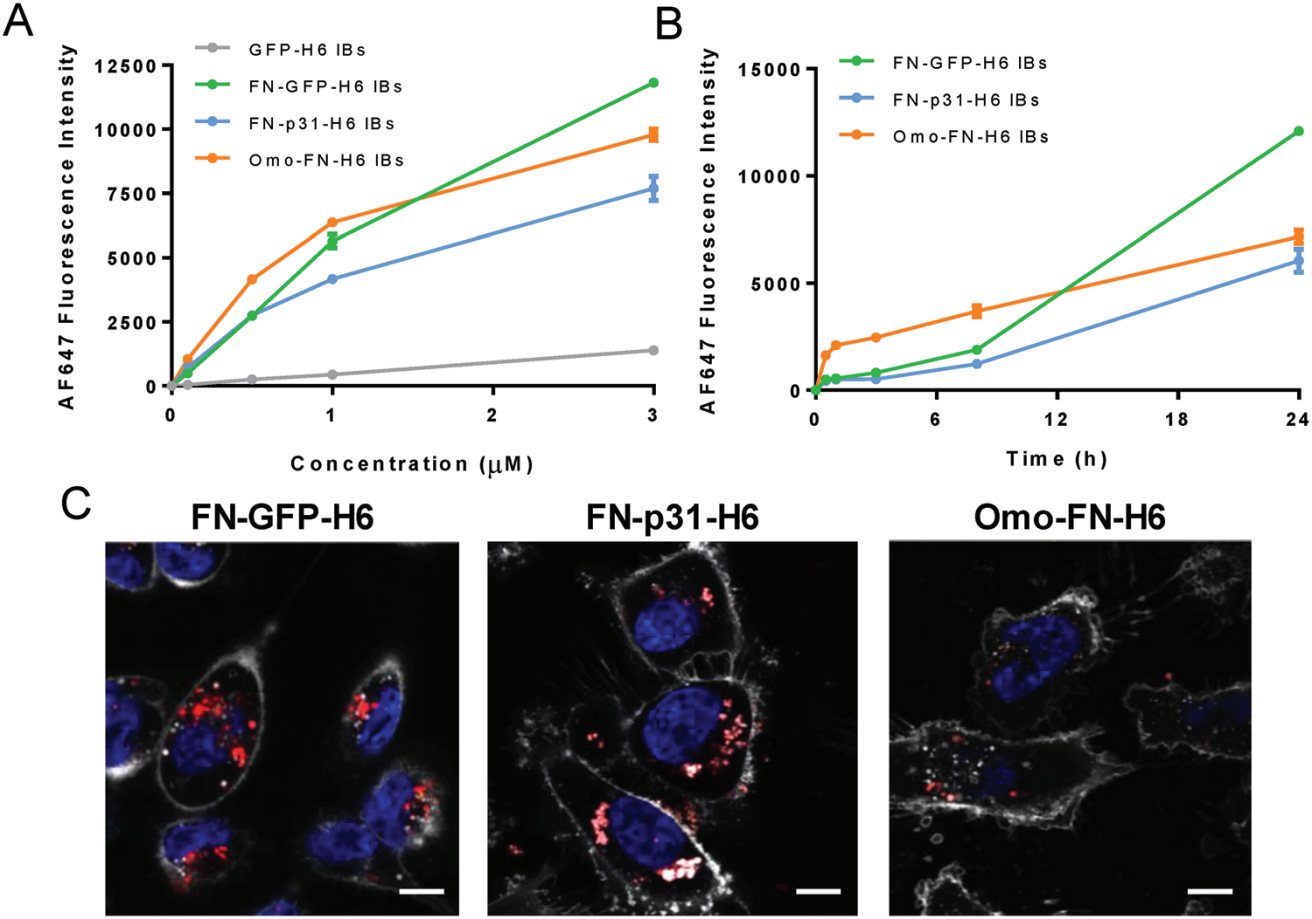
Cell penetration of CD44‐targeted IBs. A) Quantification of protein internalization by fluorescence was quantified with flow cytometry into MDA‐MB‐231 cells incubated for 24 h at increasing concentration of IBs. B) Kinetics of IB penetration into MDA‐MB‐231 cells exposed to 3 × 10^−6^ m IBs. C) Representative confocal microscopy images of protein internalization in MDA‐MB‐231 after 24 h treatment with 3 × 10^−6^ m of IBs. Nuclei and membrane cells were labeled with Hoechst (blue) and WGA (light grey) respectively. Magnification bars represent 5 µm.

Since both p31 and Omomyc perform their activities intracellularly, cell uptake of IBs can be inferred from the cytotoxicity exhibited by these materials on cultured cells, in contrast with the absence of effect shown by control GFP IBs (Figure 2). However, to effectively assess the cell penetrability of cytotoxic IBs, these materials were labeled with AF647. When MDA‐MB‐231 cells were exposed to labeled IBs, intracellular fluorescence increased in a dose‐dependent fashion when cells were exposed to FNI/II/V functionalized IBs (**Figure** **3**A). However, GFP‐H6 IBs, which lack a cell ligand, did not accumulate in CD44 target cells (Figure 3A). This confirms that cell targeting of IBs for specific protein delivery is a feasible concept, not restricted to the CXCR4‐binding T22 peptide, in which this event has been recently described for the first time.^30^ Internalization efficacy of p31‐ and Omomyc‐ based IBs was comparable (Figure 3A,B) and with similar intracellular distribution (Figure 3C). Furthermore, FNI/II/V‐functionalized IBs accumulated inside target cells overtime, a fact that is compatible with moderate or absent lysosomal degradation (Figure 3B). In this regard, the confocal analyses of the internalizing material revealed a punctuated fluorescence pattern indicative of an endosomal route of internalization, and a perinuclear localization of most of the engulfed protein particles that was more evident in the case of p31 IBs (Figure 3C).

**Figure 4:**
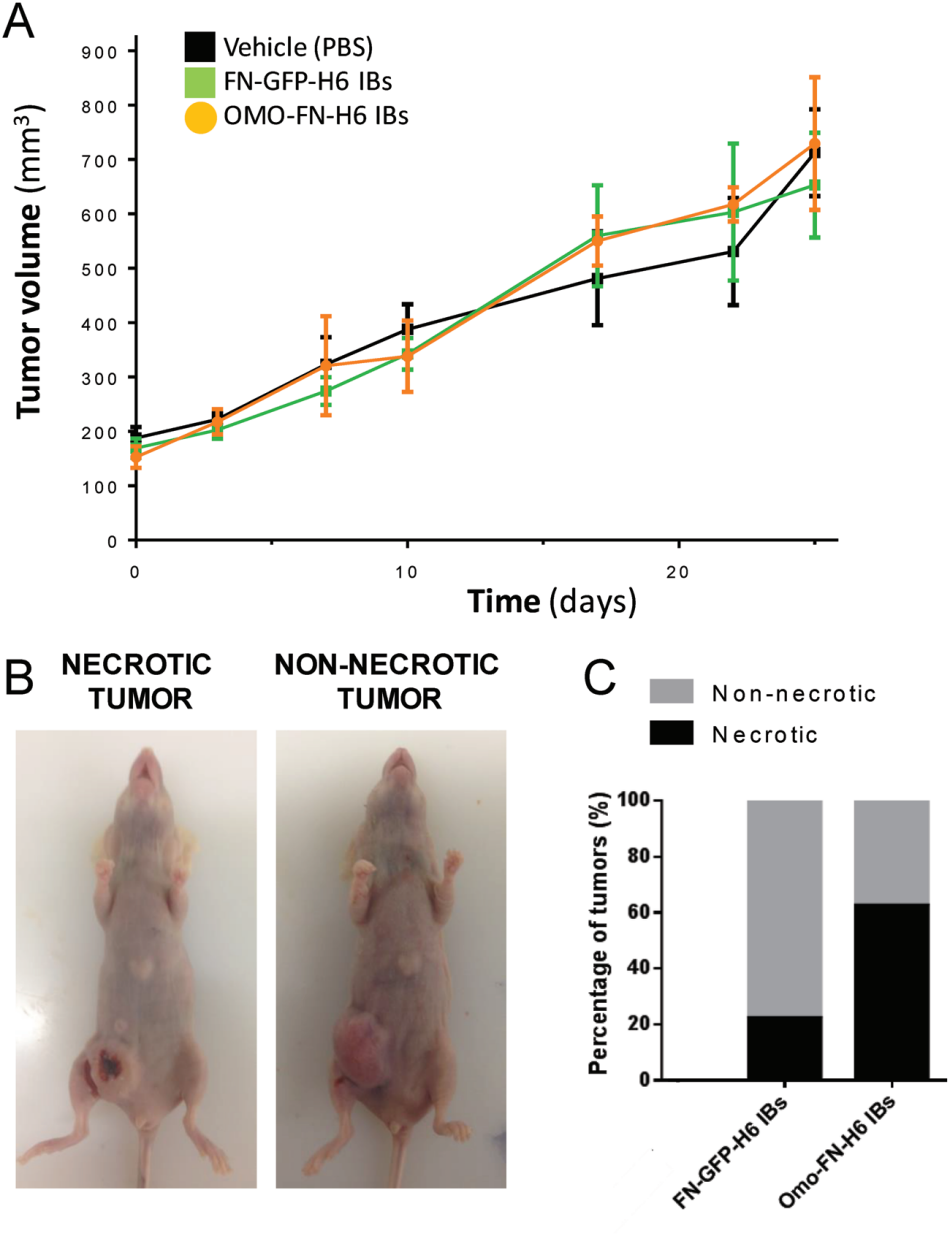
Antitumoral effect of IBs. A) Effect of Omomyc‐based IBs on tumor growth. Balb/c nude female mice bearing orthotopic tumors of MDA‐MB‐231 cell line were divided in 5 groups (*n* = 7–8) and treated intratumorally (i.t.) once a week. One group was treated with Omomyc IBs. GFP‐based IBs and PBS were included as treatment controls. No significant effect on tumor volume between the treatment groups was detected. B) Tumor necrosis was also evaluated at the experiment end point. Representative images are shown. C) The percentage of necrotic tumors for the groups treated with either IBs was measured. Omomyc IBs treated mice showed an increase in the number of necrotic tumors compared to GFP IB‐treated animals.

Because of the high penetrability capacity of CD44‐targeted IBs in cultured cells, we decided to evaluate their potential macroscopic effect in vivo, in mouse cancer models. For a first screening of antitumoral effect, we chose to test Omomyc IBs, which had shown superior cytotoxicity in vitro compared to FN‐p31‐H6. Omomyc IBs were injected intratumorally in an orthotopic animal model of human breast cancer and the biological impact of the administered materials on the tumor progression was evaluated in repeated dose administration (weekly) for 4 weeks. Although the general tumor volume measured by caliper was not significantly affected during the test period (**Figure** **4**A), the proportion of necrotic tumors (Figure 4B) was clearly higher in the group of animals receiving Omomyc IBs compared to those in control groups (Figure 4C). This observation indicated destruction of tumoral tissue, which, although not reflected by any significant change in tumor volume, was representative of a relevant biological effect. Hence, the detected antitumoral effect was indicative of the functional IBs having an impact on the biology of tumoral target cells.

**Figure 5:**
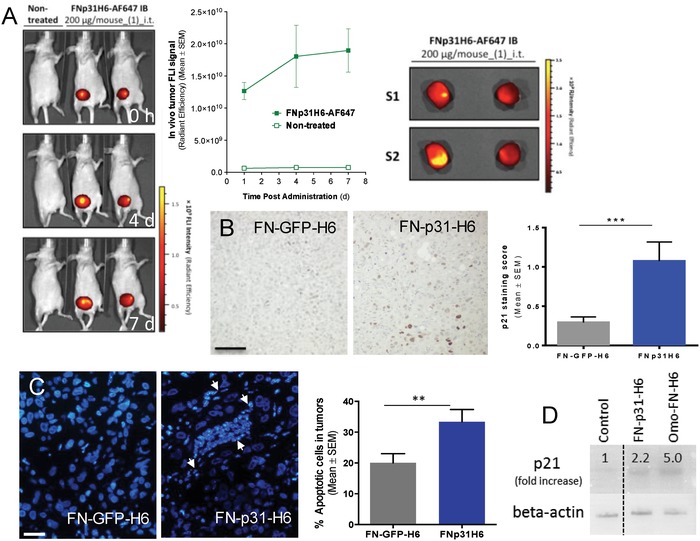
In vivo biodistribution and efficacy of FN‐p31‐H6 IBs. A) Mice bearing orthotopic tumors of HCC1806 cell line were treated intratumorally (i.t.) with p31 IBs labeled with the AF647 fluorochrome. In vivo imaging was performed with IVIS‐Spectrum 0, 4, and 7 days after administration (left panel) and quantified (right top panel). No fluorescence was observed outside the tumors (not shown). Ex vivo imaging (right bottom panel) confirmed that fluorescence was retained in the tumors, with higher intensities at the site of injection. B) Representative images of p21 protein expression by immunohistochemistry in tumors of animals treated i.t. with control GFP or p31 IBs, showing that only p31 containing IBs were able to induce p21 expression, in BT‐20 orthotropic tumors. Quantitative evaluation of the stained section confirmed that p21 staining was significantly higher in tumors of p31 IBs‐treated mice (right panel). C) Representative images of Hoechst staining of control FN‐GFP‐H6‐treated and p31 IBs‐treated mice. Arrows indicate the presence of characteristic apoptotic nuclei. Quantification of apoptotic nuclei in control and FN‐p31‐H6 treated tumors indicated that apoptosis was significantly increased upon treatment with p31 IBs (right panel). Magnification bars in (B,C) represent 100 and 50 µm, respectively. *p* < 0.01 (**); *p* < 0.001 (***). D) p21 expression in BT20 cells treated for 4 h with FN containing constructs.

In parallel, we continued the characterization of p31‐based IBs assessing their behavior in in vivo conditions. When these IBs were administered in a single dose, the fluorescent material was detected up to 7 d in the injection site, without apparent loss of emission (**Figure** **5**A), confirming the stability of the protein particles. In this context, p21 overexpression (a cell cycle regulator usually associated to growth arrest and/or senescence) was also clearly observed by immunodetection in tumors treated with FN‐p31‐H6 IBs (Figure 5B). Since the nuclear overexpression of p21 has been associated to the triggering of apoptotic cascades through the blocking of cyclin dependent kinases,^31^ the presence of apoptotic cells was assessed in BT‐20 tumors upon single dose administration of FN‐p31‐H6 IBs, since these cells were the ones with the highest cell growth inhibition in vitro (Figure 2C). As observed in Figure 5C, a significant increase in apoptotic cell bodies was clearly visible in FN‐p31‐H6 IBs treated tumors when compared with control animals, and overexpression of p21 was confirmed by Western Blot in BT20 cells exposed to both IBs (Figure 5D).

In summary, self‐assembling proteins and protein materials are of emerging interest because of their intrinsic lack of toxicity, biodegradability, functional and structural versatility, and because of their suitability for biological fabrication in a diversity of microbial cell factories.^32^ Some of them, such as the bovine α‐lactalbumin^33^ or the hen egg white lysozyme,^34^ have already proved their utility as the basis of wide spectrum antitumoral drugs. Among the diversity of applications of protein constructs, bacterial IBs have found different promising niches in biomedical research,^35^ as delivery systems of therapeutic proteins^8^, ^11^ or as convenient agents in immunoprophylaxis and immunotherapies.^36^ The utility of this platform is based on the high mechanical stability of IBs combined with the ability of the particles to release functional proteins under physiological conditions both in vitro and in vivo in cell culture media,^37^ the intracellular environment^9^ or as locally administered in organs or tissues of entire organisms.^13^ Both the mechanical stability and capability of protein release are generic IB properties^38^ supported by the particular sponge‐like architecture (combining amyloidal and nonamyloidal protein forms) of IBs.^3^ Protein released from subcutaneous IBs can reach the bloodstream and be remotely delivered to target organs, provided the IB protein incorporates appropriate ligands for cell surface receptors.^12^ In addition, IBs are also nontoxic in vitro when exposed to cultured mammalian cells^4^ or upon systemic administration (i.e., by oral delivery).^8^ Therefore, these materials are promising as implantable protein depots for the release of functional polypeptides with therapeutic value aimed to the local or generic treatment of chronic diseases. A critical step in this direction would be to generate a proof of concept of the healing potential of the biomaterials in clinical contexts.

In this regard, we have here proved for the first time that two recombinant proteins with recognized antitumoral effects, empowered with a potent ligand of the tumoral marker CD44 (Figure 1), are able to significantly impact tumor cell biology (Figures 2, 4, and 5) through the internalization of these biomaterials in target cells, both in vitro in cell culture (Figure 3) and in vivo in entire organisms (Figure 5). Indeed, we have demonstrated here that upon efficient internalization, engineered versions of p31 and Omomyc, formulated as CD44‐targeted IBs, promote selective target cell death through their respective impacts in regulatory cell circuits.

Omomyc anti‐tumor activity is based on its direct interference with Myc function. Myc has been shown to be involved in multiple aspects of tumorigenesis,^39^ both at the intracellular and extracellular level, being responsible for cell division, increased cell metabolism, immune tolerance, and survival to treatment of tumor cells. The benefit of inhibiting Myc has been demonstrated by multiple studies,^19^ which indicated that interfering with Myc function does not only lead to cell growth arrest, but also to energetic crisis, anti‐tumor immune reprogramming, and cancer cell death. Omomyc has been instrumental in demonstrating this therapeutic opportunity, first as a transgene^20^ and more recently as a therapeutic polypeptide,^40^ while also demonstrating the complete safety of the approach for normal tissues. The potential use of Omomyc as a therapeutic mini‐protein has only emerged recently^40^, ^41^ and, to date, this is the first and only report of its application in the IB format, which establishes the feasibility of its topical administration by intratumoral injection directly to the tumor site.

P31, so far neglected regarding its potential uses as a therapeutic agent, has been directly linked to the control of p21 expression and pro‐apoptotic activity, especially in triple negative breast cancers (TNBC).^42^ The present study proves that P31 is packable as IBs while keeping relevant antitumoral functionalities. This is not only pertinent regarding the incorporation of this protein to the growing catalogue of putative protein drugs, but also because it indicates that the engineering of antitumoral IBs is a general concept not restricted to a specific polypeptide. Importantly, the IB format itself is nontoxic, as CD44‐targeted GFP IBs do not cause any deleterious effects over exposed cells (Figure 2).

## 3 Conclusions

Since the biological production of IBS is cost‐effective, using this material appears to be a convenient way of local administration of protein drugs. Considering the increasing spectrum of therapeutic proteins already approved or under development for cancer therapies, most of them produced by recombinant DNA technologies,^43^ their potential packaging as IBs might largely expand their spectrum of activities and applicability in innovative and personalized medicines, for oncology and in other fields of human or animal clinics.

## 4 Experimental Section

*Protein Design, and IB Production and Purification*: Two modular proteins were designed to contain the CD44 ligand FNI/II/V fused to either p31 (the C‐term fragment of p130cas) or Omomyc (the Myc‐derived bHLHZip domain mutant), appended with a His‐tag (Figure 1). The FNI/II/V domain was placed at the N‐ or C‐terminus of p31^17^ and Omomyc‐containing modular protein respectively, considering preliminary results which showed no impact on protein activity. The amino acid sequences of the fusion proteins are MWQPPRARITGYIIKYEKPGSPPREVVPRPRPGVTEATITGLEPGTEYTIYVIALKNNQKSEPLIGRKKTGGSSRSSSGQYENSEGGWMEDYDYVHLQGKEEFEKTQKELLEKGSITRQGKSQLELQQLKQFERLEQEVSRPIDHDLANWTPAQPLAPGRTGGLGPSDRQLLLFYLEQCEANLTTLTNAVDAFFTAVATNQPPKIFVAHSKFVILSAHKLVFIGDTLSRQAKAADVRSQVTHYSNLLCDLLRGIVATTKAAALQYPSPSAAQDMVERVKELGHSTQQFRRVLGQLAAAGGSSRSSSKHHHHHH (FN‐p31‐H6) and MTEENVKRRTHNVLERQRRNELKRSFFALRDQIPELENNEKAPKVVILKKATAYILSVQAETQKLISEIDLLRKQNEQLKHKLEQLRNSCAGGSSRSSSWQPPRARITGYIIKYEKPGSPPREVVPRPRPG VTEATITGLEPGTEYTIYVIALKNNQKSEPLIGRKKTGGSSRSSSKHHHHHH (Omo‐FN‐H6) respectively. FN‐p31‐H6 and Omo‐FN‐H6 genes, together with the biologically inactive control gene FN‐GFP‐H6 and nontargeted GFP‐H6 control gene, were provided by GenScript (Hong Kong, China). Recombinant proteins were produced in *E. coli* strain BL21 (DE3), except Omo‐FN‐H6 that was produced in BL21 pLys cells. Protein production was usually induced at 37 °C for 3 h upon 1 × 10^−3^ m IPTG addition. FN‐GFP‐H6 protein was produced overnight at 16 °C. Bacterial IBs were purified upon protein production; cells were incubated 2 h at 37 °C with a Protease Inhibitor Complete tablet, 10 × 10^−3^ m PMSF and 1 µg mL^−1^ lysozyme. Then, cultures were treated with 0.1% Triton X‐100 for 1 h at room temperature (RT). Cycles of freeze/thaw were applied daily to destroy remaining bacteria until reaching CFU mL^−1^ < 10^−2^. Samples were incubated with 0.02% NP‐40 for 1 h at 4 °C followed by 1 h incubation with 1 µg mL^−1^ DNAse at 37 °C. IBs obtained after centrifugation at 15 000 *g* were washed twice with PBS, separated in 1 mL aliquots and stored at −80 °C in PBS. A milder purification protocol was applied for GFP‐H6 IBs isolation. After the incubation with PMSF and lysozyme, cells were lysed using a French Press at 1200 psi. Lysates were treated with 0.1% Triton X‐100 for 1 h at RT followed by 1 h incubation with 1 µg mL^−1^ DNAses at 37 °C. Cycles of freeze/thaw were applied daily until reaching CFU mL^−1^ < 10^−2^. IBs were centrifuged, washed twice in PBS and stored at −80°C in PBS in 1 mL samples.

*IB Protein Quantification*: Serial dilutions of 1 mL IB samples were prepared in water with Laemmli buffer and incubated at 98 °C for 45 min. IB dilutions together with a soluble GFP‐H6 standard of known concentration were loaded on SDS PAGE. Protein quality was analyzed by Coomassie blue staining, while protein quantification was calculated by Western Blot using an anti‐His antibody (sc57598, Santa Cruz Biotechnology, Santa Cruz, CA, USA). Protein bands were quantified from the standard curve fitting equation of GFP‐H6 using the Quantity One software.

*Cell Viability Assay*: Cell viability of breast cancer cell lines (MDA‐MB‐231, MDA‐MB‐468, BT‐20, BT549, and HCC1187) upon treatment with IBs was measured by the MTT metabolic test (Roche, Basel, Switzerland) following manufacturer recommendations. MDA‐MB‐231, MDA‐MB‐468, BT‐20, BT549, and HCC1187 cells were plated at 2500 c/well, 5000 c/well, 6000 c/well, 1000 c/well, and 6000 c/well respectively for 24 h. Cells were incubated with 0.5, 1, and 3 × 10^−6^ m of IB protein. Viability was measured at 48, 72, and 96 h comparing luciferase signal against untreated control cells.

*MDA‐MB‐231 Mammosphere Production*: MDA‐MB‐231 cells were seeded at 1000 c/well in low attachment cell culture plates (Nunc, Waltham, MA, USA) and cultured in a defined media without serum^44^ at 37 °C and 5% CO_2_. After 5 and 7 d of incubation mammospheres appeared and cultures were challenged by the addition of 3 × 10^−6^ m FN‐GFP‐H6 and FN‐p31‐H6 IB proteins and Omo‐FN‐H6 IB protein for 72 h. Sphere integrity and morphology were assessed by bright field imaging in an Olympus BH2 microscope (Olympus Corporation, Tokyo, Japan).

*Field Emission Scanning Electron Microscopy*: To characterize the morphometry (size and shape) at ultrastructural level of IBs at nearly native state, a rapid and easy method was used. Microdrops of 5 µL of diluted samples were deposited for 2 min on silicon wafers (Ted Pella Inc, Redding, CA, USA), liquid excess was blotted, air dried and immediately monitored without coating in a Field Emission Scanning Electron Microscope (FESEM) Zeiss Merlin (Carl Zeiss, Oberkochen, Germany) operating at 1 kV. IB images were collected with a standard secondary electron (SE) detector (4000–40,000x magnifications).

*IB Labeling*: AlexaFluor 647 NHS (Molecular Probes, Eugene, OR, USA) was used to label the inclusion bodies so that internalization assays could be normalized and compared at a unique wavelength. Conjugation was performed by resuspending IBs at 1 mg mL^−1^ in PBS with the addition of dye at a dye:protein molar ratio 2:1 and incubating for 1 h at RT with agitation. Serial cleaning steps were applied to remove free dye by centrifuging and resuspending the IBs with H_2_O. Fluorescence units/mass was determined by flow cytometry.

*Confocal Laser Scanning Microscopy*: MDA‐MB‐231 cells were seeded on Mat‐Teck culture plate (Mat Teck Corporation, Ashland, MA, USA) at 200 000 c/well for 24 h. Medium was removed and changed to OptiPRO serum‐free medium supplemented with L‐glutamine and 1 × 10^−6^ m of IBs was added and incubated for 24 h. After incubation, cells were washed with PBS and 3 µg mL^−1^ WGA 594 (Molecular Probes) and 0.2 µg mL^−1^ Hoechst 33 342 (Molecular Probes) were added for 5 min in darkness to visualize the plasma membrane and the nuclei respectively. Later, cells were washed with PBS and complete medium was added. Stained cells were examined using a TCS‐SP5 confocal laser scanning microscope (Leica Microsystems, Heidelberg, Germany) with a Plan Apo 63 × /1.4 (oil HC × PL APO l blue) objective. Hoechst 33 342, WGA 594 and Alexa Fluor 647 were excited by a blue diode (405 nm), a helium–neon laser (594 nm) and a helium–neon laser (633 nm) respectively. Z‐series were collected at 0.5 mm intervals. Images were processed using Imaris version 6.1.0 software (Bitplane, Zürich, Switzerland).

*Flow Cytometry*: MDA‐MB‐231 cells were cultured on a 24 well plate at 80 000 c/well and incubated at 37 °C and 5% CO_2_ in a humidified atmosphere for 24 h. Cells were incubated with IBs at increasing concentrations and incubation times (internalization kinetics). After protein incubation, cells were treated with 1 mg mL^−1^ trypsin for 15 min to detach cells and remove unbound protein, followed by the addition of complete medium and centrifugation at 1400 *g* for 5 min. Collected cells were then resuspended in DPBS. Protein internalization was analyzed using a FACS‐Canto system (Becton Dickinson, Franklin Lakes, NJ, USA), with a 15 W air‐cooled red diode laser at 635 nm excitation. Alexa fluor 647 emission was measured with an FL4 detector (661/16 nm band pass filter). CD44‐positive cells were determined as described.^45^

*Human Cancer Animal Models*: All the animal studies were performed in accordance with the ARRIVE guidelines and the 3 Rs (rules of Replacement, Reduction, and Refinement). Mice were housed and treated following the protocols approved by the Ethical Committee for the Use of Experimental Animals (CEEA) at the Vall d'Hebron Research Institute (VHIR), Barcelona. MDA‐MB‐231 cells, classified as mesenchymal‐like,^46^ were resuspended in cold PBS at 15 000 000 c mL^−1^ and maintained on ice. Before surgery, mice were anesthetized with 2% isoflurane and buprenorphine (0.75 mg kg^−1^) was administered subcutaneously. 100 µL (1 500 000 c/mouse were injected between the fourth and fifth right mammary fat pads of 8‐week‐old BALB/c nude female mice (*n* = 40). Tumor size was evaluated 2 times a week by caliper measurements and tumor volume calculated using the following formula: volume = (*D* × *d* ^2^) / 2, where “*D*” is the largest diameter and “*d*” the smallest one. Weight of the mice was measured at least twice a week. When tumors reached a volume between 100 and 350 mm^3^, animals were randomized in the different groups (*n* = 7–8): control buffer (50 × 10^−3^ m KH_2_PO_4_, 500 × 10^−3^ m NaCl, 0.8 m Urea, 100 × 10^−3^ m GuHCl; pH 7.4), PBS, FNI/II/V‐GFP‐H6, and Omo‐FNI/II/V‐H6. Protein stocks were diluted to 4 g L^−1^ for the treatments. Animals were treated intratumorally once a week and the volume/dose of intratumoral administration was adjusted according to tumor volume (1:10 of the tumor volume was inoculated up to a maximum of 50 µL, e.g. a tumor of 220 mm^3^ was treated with 22 µL). Animals were treated up to 4 weeks, then euthanized by CO_2_.

For biodistribution and efficacy studies, exponentially growing HCC1806 (2.5 × 10^6^) or BT20 (10 × 10^6^) cells, respectively, were orthotopically implanted into the fat mammary pad (i. f. m.p.) and monitored weekly as described above. These cells are classified as basal like^47^ and luminal androgen receptor (LAR),^48^ respectively. In biodistribution assays, once the tumors reached an average size of 150 mm^3^, mice were intratumorally administered with Alexa 647 labeled FN‐p31‐H6 IBs (200 µg per mouse, *n* = 3) and fluorescent signal was monitored in vivo at different time periods post‐administration by means of IVIS Spectrum equipment (Perkin Elmer, Tres Cantos, Spain). Quantification of the fluorescent signal (in Radiant Efficiency) was performed by using the Living Image software (Perkin Elmer). At the end point, tumors were excised and subjected to fluorescent imaging. Afterward, tissues were snap frozen and stored for further analysis or fixed in 4% formaldehyde and processed for histopathological analysis and evaluation. For efficacy assays, mice bearing BT20 tumors with an average size of 200 mm^3^ (*n* = 8), were administered with 600 µg of FN‐p31‐H6 or FN‐GFP‐H6 IBs. Administration was repeated twice in two week and animals were euthanized one week after the last treatment.

*Tumor p21 Immunohistochemistry and Apoptosis*: The presence of p21 antigen in tumor sections was analyzed by pre‐treating paraffin embedded sections with 100 × 10^−3^ m citrate buffer (pH 9) in a cooker. Sections were incubated with 10% normal goat serum (NGS) in antibody diluent (1% BSA in 100 × 10^−3^ m Tris buffer) and then of 1:100 dilution of anti‐p21 antibody (M 7202, Dako, Santa Clara, CA, USA). Secondary antibody consisted in a HRP conjugated system (EnVision+ System‐HRP Labeled Polymer anti‐Mouse), which was later visualized with DAB and counterstained with Harris haematoxylin. The p21 signal intensity and extension were scored under the light microscope by two blinded observers. Intensity cores ranged from 0 to 3 (absence, low intensity, normal or high, respectively). For each tumor, two independent sections with p21 staining were evaluated. For the evaluation of the extension of the apoptotic nuclei, sections were stained with Hoescht (1:500, Sigma‐Aldrich, San Luis, MI, USA) mounted in Prolong (Invitrogen, Carlsbad, CA, USA). Two independent slides per tumor and five representative images per slide were analyzed after acquiring images at 10x magnification in an Olympus BX61 microscope (Olympus Corporation). In each image, the number of apoptotic nuclei versus the number of nonapoptotic nuclei was counted by two independent blinded observers. Results were presented as percentage of apoptotic nuclei. Anti‐p21 (MS‐891‐B Thermo Scientific) Western Blot was performed on cell extracts as described^49^ using BT20 cells exposed to 9 × 10^−6^ m protein for 4 h. Fold increase of p21 was referred to a β‐actin control.

*Statistical Analysis*: Quantitative data were expressed as mean and standard error (x̄ ± SE). Depending on the type of data, parametric or nonparametric statistics were applied to compare divergences between all groups in relation to control or pairwise comparisons. In some data, one‐way ANOVA followed by a post‐hoc Dunnett was used, whereas Kruskall–Wallis and Mann Whitney or Wilcoxon tests were performed with the rest of data. Statistical significance levels were represented as n.s. *p* > 0.05, * *p* < 0.05, ** *p* < 0.01, and *** *p* < 0.001. All analyses were performed using SigmaPlot 10.0 software.

## Conflict of Interest

T.J. is an employee of Peptomyc S.L.; L.S. and M.E.B. are co‐founders and shareholders of the same company.
